# Digitalis in Pneumonic Fever

**Published:** 1897-05

**Authors:** E. V. Ross

**Affiliations:** Rochester, N. Y.


### DIGITALIS IN PNEUMONIC FEVER.

E. V. Ross, M. D., Rochester, N. Y.

In the so-called senile pneumonia or the pneumonia occurring in old people, Digitalis is a potent remedy when the following set of symptoms is present:

"Dry cough, with mucus râles over both lungs, without expectoration; if there be expectoration it has a purplish color, which has been likened to 'prune juice.' "

"Face pale, of a death-like appearance, or a purplish cyanotic hue."

"Extremities cold and cyanosed."

"Pulse feeble, frequent, irregular, and may intermit."

"Great prostration."

"Deathly nausea, or a gone, sinking feeling in the scrobiculus cordis."

The above symptoms indicate a desperate condition, and point quite clearly to an impending respiratory paralysis. The prune-juice expectoration, so characteristic of Digitalis, is looked upon as an unfavorable sign, indicating extensive blood changes. According to Grisolle* the mortality in pneumonia is 59 per cent. in those over sixty years of age.

Antimonium-tart. is the nearest analogue in these cases.

